# Chemiresistors with In_2_O_3_ Nanostructured Sensitive Films Used for Ozone Detection at Room Temperature

**DOI:** 10.3390/gels9050355

**Published:** 2023-04-23

**Authors:** Mariana Chelu, Paul Chesler, Cristian Hornoiu, Mihai Anastasescu, Jose Maria Calderon-Moreno, Daiana Mitrea, Costin Brasoveanu, Carmen Moldovan, Mariuca Gartner

**Affiliations:** 1 “Ilie Murgulescu” Institute of Physical Chemistry—Romanian Academy, Splaiul Independentei 202, 060021 Bucharest, Romania; 2 National Institute for Research and Development in Microtechnologies, Strada Erou Iancu Nicolae 126A, 077190 Voluntari, Romania

**Keywords:** In_2_O_3_ nanostructured films, sol-gel, Au IDEs, Pt heater, ozone detection, room temperature

## Abstract

Detection of greenhouse gases is essential because harmful gases in the air diffuse rapidly over large areas in a short period of time, causing air pollution that will induce climate change with catastrophic consequences over time. Among the materials with favorable morphologies for gas detection (nanofibers, nanorods, nanosheets), large specific surfaces, high sensitivity and low production costs, we chose nanostructured porous films of In_2_O_3_ obtained by the sol-gel method, deposited on alumina transducers, with gold (Au) interdigitated electrodes (IDE) and platinum (Pt) heating circuits. Sensitive films contained 10 deposited layers, involving intermediate and final thermal treatments to stabilize the sensitive film. The fabricated sensor was characterized using AFM, SEM, EDX and XRD. The film morphology is complex, containing fibrillar formations and some quasi-spherical conglomerates. The deposited sensitive films are rough, thus favoring gas adsorption. Ozone sensing tests were performed at different temperatures. The highest response of the ozone sensor was recorded at room temperature, considered to be the working temperature for this specific sensor.

## 1. Introduction

Stratospheric ozone exists naturally in the upper atmosphere and protects us from damage caused by the sun’s ultraviolet rays, having a beneficial effect on the planet [1].

Tropospheric ozone (ground-level ozone or surface ozone), hereafter referred to as ozone (O_3_), is a toxic air pollutant that can harm human health and the environment, being the main component of “smog” [2]. O_3_ is a secondary pollutant that is produced when primary pollutants, such as volatile organic compounds (VOCs) and nitrogen oxides (NO_x_) from stationary air, chemically react in the presence of sunlight [3]. When the level of pollutants emitted by automobiles, refineries, power plants, industrial facilities or factories is very high during sunny periods, there is a very high level of ozone in those areas. It can also have a negative impact on vegetation and ecosystems [4,5].

Breathing atmosphere containing high levels of ozone affects certain individuals, such as asthmatics, the elderly, children and those who perform extended outdoor activities. For this reason, OSHA (Occupational Safety and Health Administration) recommends a human exposure threshold of 0.1 ppm O_3_ in air for a maximum of 8 h, as higher concentrations (>50 ppm) can cause serious illness (both healthy adults and asthmatics may experience significant reduction in lung function and inflammation of the upper airways) or even death. At the same time, the European Air Quality Directive 2008/50/EC sets a target value of 120 µg/m^3^ O_3_ for the average daily concentration of 8 h of exposure, which can be exceeded up to a maximum of 25 days per year on average over 3 years [6,7]. An extensive study analyzed the high concentrations of surface ozone in the area of Bucharest, the capital of Romania, using both data measured at monitoring stations and data obtained from satellites. These were correlated with high NO_x_ emissions due to road traffic in the urban area of the capital, compared to other regions of the country [8]. Consequently, to effectively control pollutant emissions, reliable and accurate air quality data are needed. In this sense, there is an increased interest in the development of high-performance air quality monitoring systems, based on low-cost sensors, which are miniaturized and easy to implement in portable devices [9].

Among various sensor systems for the detection of gaseous pollutants, metal oxide semiconductors (SMOX) are the most studied materials as O_3_ sensors, having detection limits in line with OSHA recommendation [10]. The operating principle of chemiresistive gas sensors (chemiresistors) is based on the variation of the resistance of the SMOX sensitive layer, depending on the concentration of the target gas in the analyzed atmosphere, which generates a proportional current flow in the detection device circuit [11,12,13]. Several types of SMOX have been comprehensively investigated for O_3_ detection [14,15,16,17]. For example, commercial sensors contain sensitive layers in the form of thick or thin films of indium and tin oxide (ITO), tungsten oxide (WO_3_), zinc oxide (ZnO) or titanium dioxide (TiO_2_) [18,19,20]. They are generally manufactured by sputter deposition or other high-cost methods, involving high energy consumption or expensive laboratory equipment [21,22,23,24,25]. In addition, the influence of the environment (temperature, humidity or interfering chemical species) contributes to a variation in the performance of these commercial sensors, in terms of the collected data [26].

Recently, different types of sensors have been reported for O_3_ detection that operate at a temperature lower than 100 °C with good sensor response [27,28,29,30]. Based on the density functional theory, theoretical studies were made on the interaction of the O_3_ molecule with group III nitride as well as Al and Ga doped B_12_N_12_. Exploration of the structural, thermodynamic, electronic, dipole moment and NBO (natural born orbital) charge transfer properties revealed a promising sensing behavior for the O_3_ molecule [31]. Using a simple one-step solution combustion method, *p*-type CuCo_2_O_4_ spinel nanomaterials rich in oxygen vacancies were synthesized. The CuCo_2_O_4_-based O_3_ sensor was investigated at a temperature of 90 °C, and showed a high response (R_s_ = 27)—for 1 ppm O_3_ under 70% relative humidity (RH), together with sensor chemical stability and very good sensor selectivity [32].

For monitoring tropospheric ozone in ambient air, two different types of SMOX sensors based on nanostructured sensing layers of ZnO:Ga, pure (SnO_2_) and graphene-doped tin dioxide (SnO_2_:G) nanofibers were manufactured by magnetron sputtering and electrospinning. The materials were tested and compared with commercial SMOX sensors under both controlled laboratory conditions and real-life conditions. Promising results were obtained, which showed that the prepared sensors performed similarly to the commercial sensors and exhibited excellent performance compared to the usual commercial sensors [33].

The highly sensitive photoluminescence emission of ZnO nanoparticles (NPs) at room temperature was explored for O_3_ detection. Both commercially available luminescent ZnO NPs and ZnO/polymer nanohybrids were investigated. Polymer matrices of the type poly (poly (ethylene glycol) methyl ether methacrylate) (PPEGMA) and polydimethylsiloxane (PDMS) were used. The materials were investigated by exposure to ozone gas at room temperature over a range of concentrations between 1600–50 ppb. The ZnO/PDMS-based material showed an ozone detection limit of 50 ppb, for a response/recovery time in the range of 50–100 s [34].

In this study, we report the fabrication of a room temperature-operated sensor for ozone detection, fabricated by a simple/low-cost strategy based on the preparation of the sensitive layer of In_2_O_3_ using the sol-gel method. The versatility and advantages of this ecological method consist in the ease of synthesizing the sensitive material with controlled morphology, favorable for the detection of gaseous ozone under laboratory conditions. The results presented in this paper proved the detection performance of the obtained sensor, which exhibited a limit of detection (LOD) at room temperature of 0.08 ppm O_3_. The response/recovery time of the sensor was fast; sensor signal has low-noise; and sensor recovery was complete after each sensing test, allowing for a new sensing cycle without sensor replacement.

## 2. Results and Discussions

### 2.1. Sensor Characterization Methods

#### 2.1.1. Atomic Force Microscopy (AFM)

Atomic force microscopy (AFM) measurements were performed in “non-contact” mode with an XE-100 device from Park Systems in order to minimize the probe (tip)–surface interaction. The XE100 microscope is equipped with decoupled XY and Z scanners, thus eliminating unwanted artifacts (bow effect, for example). All AFM measurements were made with NCHR tips (Nanosensors™), having a typical radius of curvature of ~8 nm, length of ~125 µm, width of 30 µm, spring constant of ~42 N/m and resonance frequency of ~330 kHz. After recording, the images were processed with the XEI program (v 1.8.0), produced by the same company (Park Systems).

The AFM figures are presented in the so-called “enhanced contrast” mode, in which the color of a pixel is determined by its variation in comparison to the neighboring pixels. Therefore, unlike the classic color mode used in AFM imaging, where the gradient of a single color is used, in the “enhanced contrast” mode, there is not a linear dependence in color with the height difference. On the other hand, the information of the profile lines (line scans) is not perturbed in the *z*-axis; instead, the morphology of the sample can be better visualized (enhanced) in the case of flat or rough samples.

**Figure 1:**
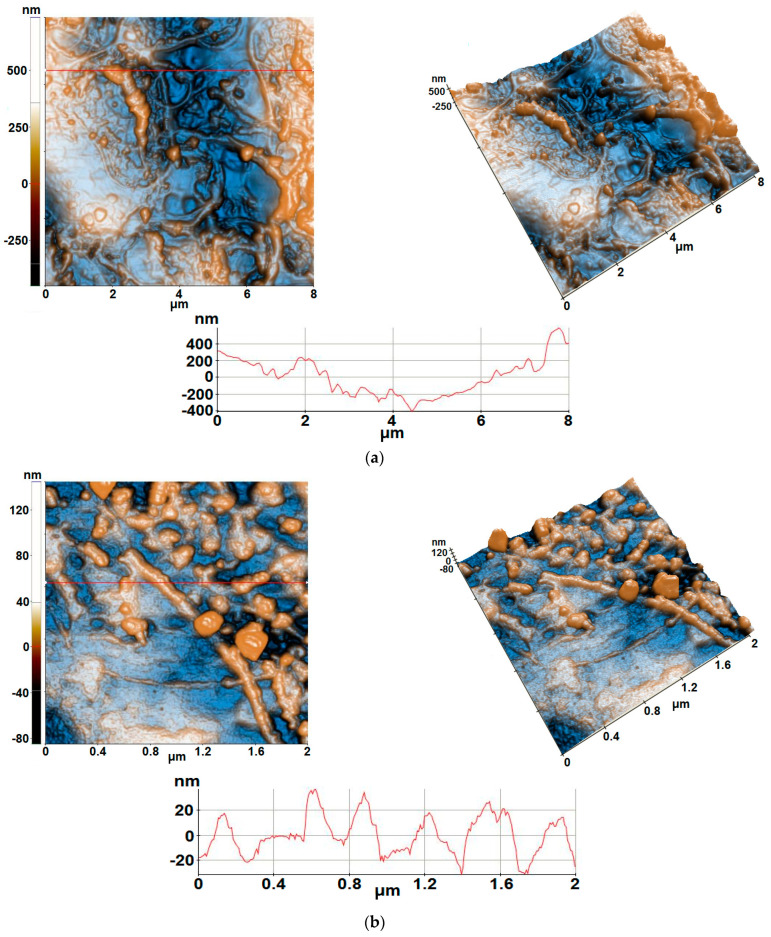
AFM images of the 10-layered In_2_O_3_ sensitive film deposited on Au-IDE/Alumina (alumina ceramic support with interdigitated gold electrodes) at the scale of: (**a**) (8 × 8) µm^2^; (**b**) (2 × 2) µm^2^.

Figure 1 presents AFM images of the sample tested from the point of view of sensor activity: a film of In_2_O_3_ obtained by 10 repetitive depositions on Au-IDE/Alumina. From Figure 1 it can be observed that the In_2_O_3_ film having 10 layers deposited onto alumina substrates (alumina transducers) is continuous, adherent to the substrate and has a complex morphology, showing the presence of both fibrillar formations and quasi-spherical conglomerates on the surface. The profile line in Figure 1a reflects a large difference in height, of about 1 μm, probably also influenced by the corrugation of the alumina ceramic substrate (which exhibit large particles on the surface—not shown here).

Thus, at the scale of (8 × 8) µm^2^ the In_2_O_3_/alumina sample exhibits a RMS (Root Mean Square)–roughness (a representation of surface roughness, calculated as deviations from the mean plane within the sampling area, using measured microscopic peaks and valleys) of 181 nm and a peak-to-valley parameter of 1185 nm. At smaller scales, scanned over (2 × 2) µm^2^, there are smoother parcels/areas, as highlighted by the profile line from Figure 1b, which shows a difference in z axis of about 60 nm and an increased repeatability/uniformity. Globally, at the scale of (2 × 2) µm^2^, a RMS roughness of 19.6 nm and a peak-to-valley parameter of 229 nm were estimated.

#### 2.1.2. Scanning Electron Microscopy (SEM)

Scanning electron microscopy (SEM) revealed the microstructure and distribution of the active phases deposited as films on the transducers, both on the alumina wafer and on the gold interdigital electrodes. The microstructure of the samples was evaluated by SEM using a Quanta 3D field emission microscope equipped with an energy dispersive X-ray (EDX) spectrometer, operating at voltages between 10 and 30 kV and at a voltage of 5 kV using secondary electron (SE) or backscattered electron (BSE) images.

**Figure 2:**
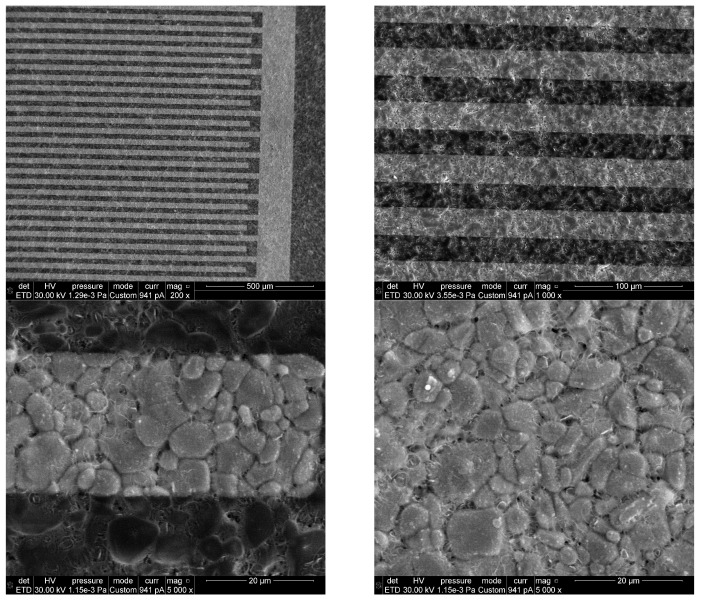

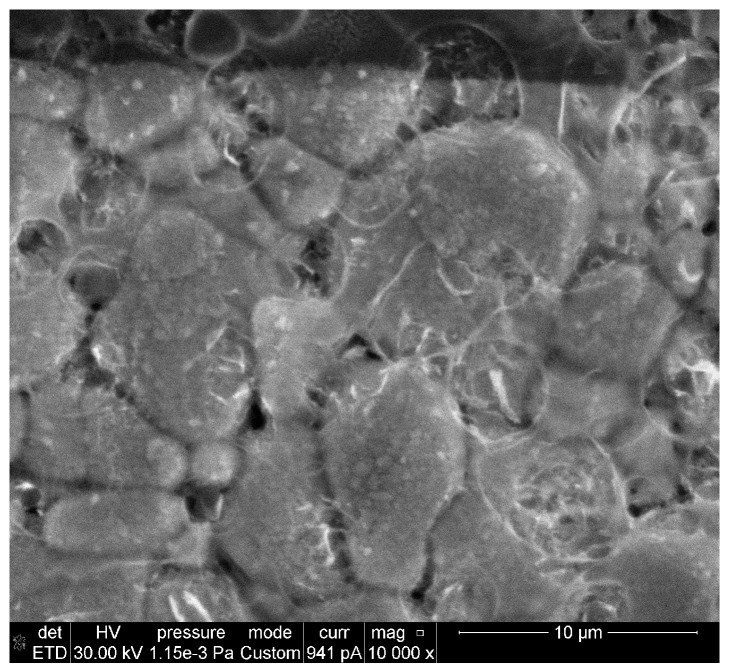
Top view SEM micrographs of the 10-layered In_2_O_3_ sensitive film of the fabricated sensor (having alumina wafer with Au IDEs), at different magnifications.

Figure 2 shows the transparent In_2_O_3_ film (gold IDEs are visible through the film) deposited on the alumina transducer. It can be seen that through successive sol-gel depositions followed by subsequent heat treatment, both the alumina substrate grains and the interdigitated Au electrodes were completely covered with a film of In_2_O_3_. Another observable feature is that the In_2_O_3_ film is continuous and folds to the characteristic surface morphology of the polycrystalline support with micron-sized alumina grains. After the deposition of the In_2_O_3_ film, the surface morphology becomes more complex, also evidenced by the AFM images, with fibrillar formations as well as some quasi-spherical conglomerates. These morphological features are probably the result of the release of gas bubbles during the drying of the sensitive layer prepared by sol-gel and heat treatment.

**Figure 3:**
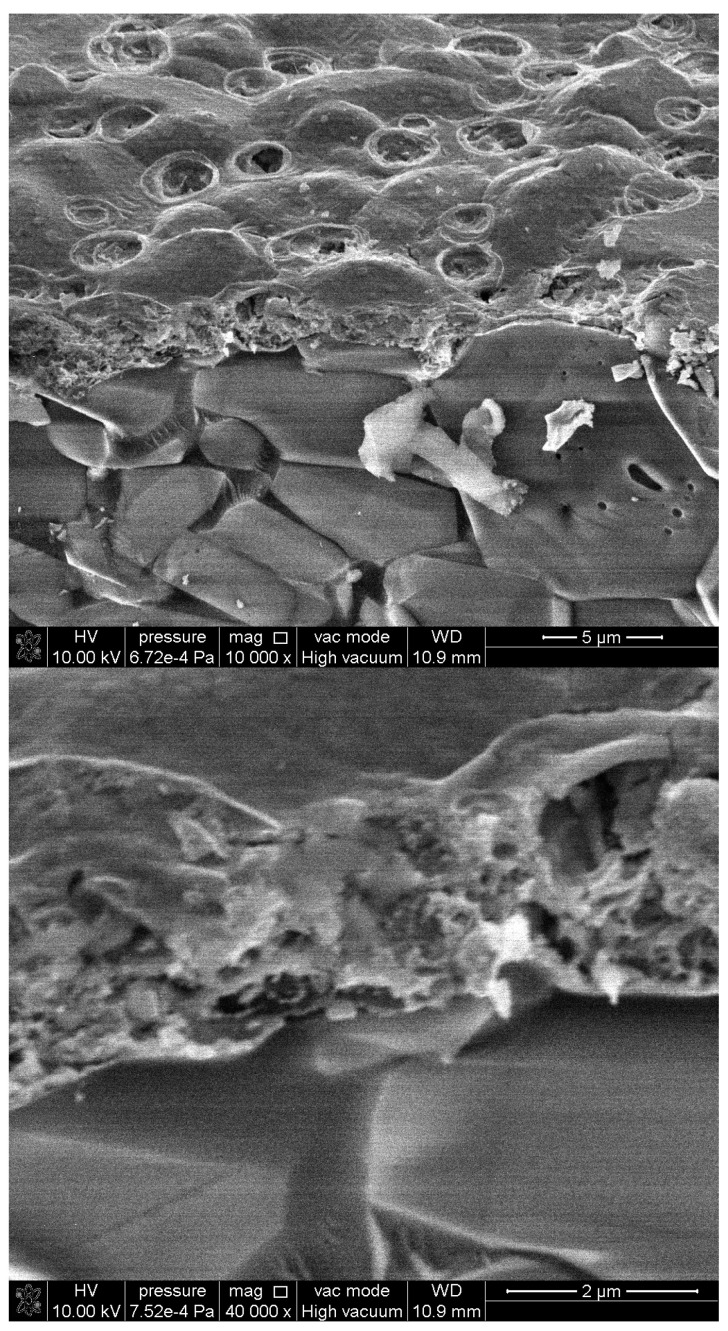
Cross-sectional SEM micrographs of the sensor’s In_2_O_3_ sensitive film at different magnifications.

Cross-sectional SEM images (Figure 3) of a fractured sample show that the In_2_O_3_ film is about 2 microns thick, yet the film conforms to the roughness of the alumina layer, which consists of alumina grains of several microns. The film is continuous, filling the intergranular step spaces and the voids between the alumina grains; therefore, the film is not of uniform thickness and can be considerably thicker at intergranular voids. The characteristics of the substrate-film interface can be observed in the cross-sectional images in Figure 3, which show close contact between the alumina substrate and the In_2_O_3_ film. The presence of porosity is clear in the cross-sectional SEM images—two types of pores exist: a fine internal porosity, which is observed throughout the cross-section and larger rounded pores on the surface with diameters similar to the thickness of the film.

**Figure 4:**
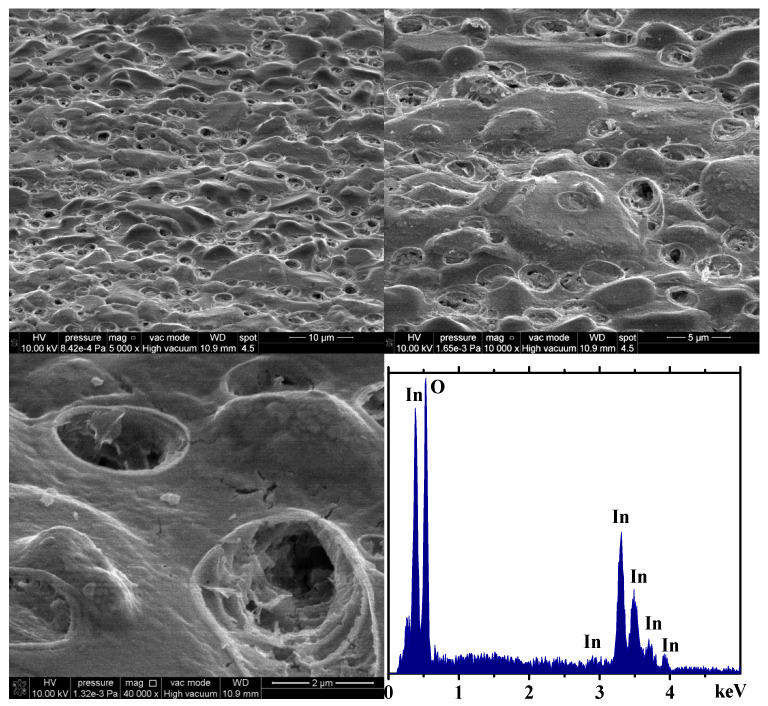
Tilted view SEM micrographs of the sensor’s 10-layered In_2_O_3_ sensitive film at different magnifications, together with EDX spectra.

Higher magnification SEM micrographs taken in tilted view of the In_2_O_3_ film (Figure 4) show the surface features and the distribution of rounded pores along the film surface. The EDX spectra (Figure 4) show only the presence of indium and oxygen. The absence of carbon indicates that the organic parts were removed during the heat treatment. The rounded-shape pores observed on the surface of the films indicates the formation of gas bubbles during the heat treatment and densification of the In_2_O_3_ film. SEM and EDX observations confirm the absence of any carbon-containing residues in the film. It should be noted that the formation of the film takes place through 10 successive depositions and, therefore, 10 successive intermediate thermal treatments, followed by a final heat treatment at a slightly higher temperature. We have used these thermal treatment conditions to remove any remaining acetylacetonate from the film, in accordance with the literature [35,36] and also with our previous studies.

#### 2.1.3. X-ray Diffraction (XRD)

**Figure 5:**
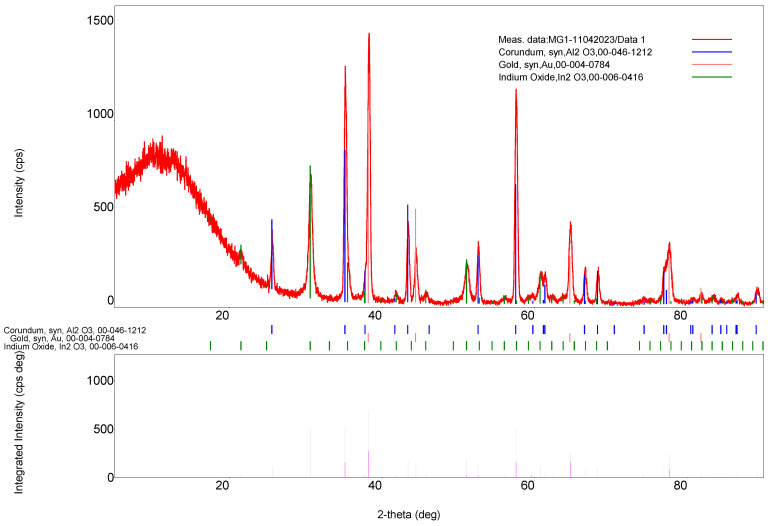
XRD pattern of the sensor sample.

X-ray Diffraction (XRD) was used to examine the structure of the sensitive film. A Rigaku Ultima IV X-ray diffractometer was used to acquire the diffractograms. The equipment was set in thin film geometry, with parallel beam (PB) optics, operated at 40 kV and 30 mA, using Cu Kα (λ = 1.5406 Å) radiation. Measurements were performed in continuous scan mode, with a scan speed of 2°/min and a step width of 0.02° (2θ), at a fixed incidence angle, α, of 0.5°. Data were collected over the angular range of 15–80° (2θ). To determine the structure of the sensor sample, X-ray diffraction was used (Figure 5).

Three compounds, α-Al_2_O_3_, Au and In_2_O_3_, were identified in the X-ray diffractogram. α-Al_2_O_3_ and Au belong to the sensor substrate (transducer), only In_2_O_3_ lines being assigned to the sensitive film. In_2_O_3_ was identified using the ICDD file no. 6-0416. In_2_O_3_ crystallized in a cubic crystal system, with space group Ia3. The lattice constant of the cubic In_2_O_3_ crystals, calculated from the whole pattern fitting, was 10.120(4) Å, very close to the indexed value (10.1180 Å in the ICDD file no. 6-0416). The crystallite size was around 17 nm, as determined by applying the Scherrer formula for the crystal plane (222) only.

### 2.2. Gas Sensing Measurements

The gas sensing experiments were performed using own-design sensing cell and transducers. The transducer model was used previously by our research group to detect small concentrations of dangerous gases [37,38]. The alumina transducer sized at 20 mm × 10 mm × 0.2 mm contains Au interdigital electrodes (IDE) with 53 pairs of interdigits, 6400 μm in length, 25 μm wide, with 25 μm spacing gap and a Pt heating circuit (1200 μm width).

**Figure 6:**
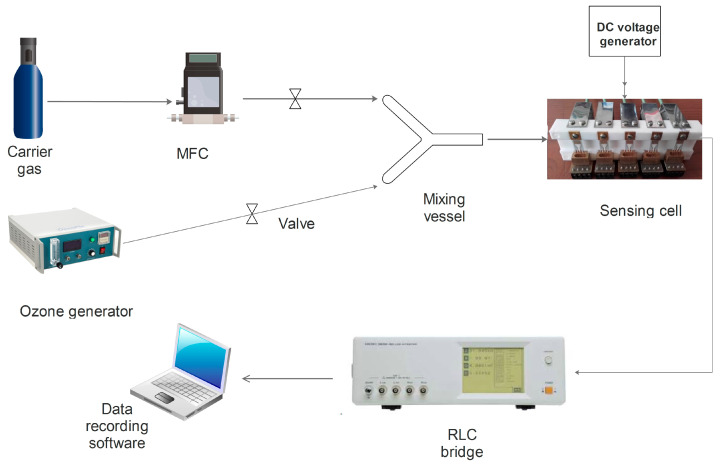
Experimental setup used for ozone sensing measurements.

To provide the target gas (different concentrations of ozone), an ozone generator was coupled with the setup gas lines (Figure 6), using a glass mixing vessel, which combines the target and the carrier gas before sensing cell entry. A commercial Dräger X-am 5000 gas detector was used to check the ozone concentration in air at the exit of the mixing vessel. The sensing measurements were performed in triplicates under laboratory conditions (the gas cylinders contain high purity dry gases, as provided by the manufacturers). To ensure the necessary working temperature, a direct current voltage generator was used to provide stable current for the contact pads of the platinum heater circuit on the back side of the sensor. The sensor operating voltage applied to the IDE pads was set at 1.5 V direct current (DC).

The fabricated sensors were tested for ozone detection in the 0.08–1.0 ppm range, considering OSHA recommendations for ozone exposure in the work environment [39], higher ozone concentrations having inflammatory effect over the human airways, as mentioned in the Section 1. The tested working temperatures for the fabricated sensors were situated in the range between room-temperature and 100 °C, because above 105 °C ozone is known to detonate [40]. Sensor response to the target gas (ozone) was defined as the ratio between sensor electrical resistance in the target gas and carrier gas (air), and it was permanently monitored and automatically recorded using a GPIB interface connected to the RLC bridge and the data acquisition computer. No additional signal filtering was involved in the sensing measurements.

**Figure 7:**
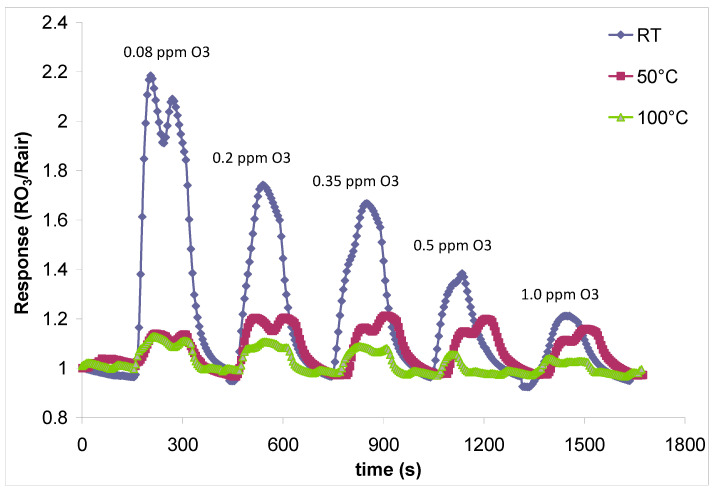
Sensor response/recovery characteristics for ozone detection at different tested concentrations and working temperatures.

From Figure 7, it can be observed that with increasing working temperatures, the response of the sensor for ozone decreases, in accordance with those mentioned above (the ozone molecule destabilizes with the increase in the temperature, until the total decomposition by explosion at 105 °C). The highest sensor response was recorded for the tested concentration of 0.08 ppm, decreasing in intensity with the increase in the concentration of the target gas (towards 1.0 ppm). At the maximum tested concentration of 1.0 ppm, the sensor generates the lowest response for ozone. Considering the room temperature testing conditions, the decrease in the sensor response with increasing tested ozone concentrations can be explained as follows: the adsorption centers on the surface of the sensitive layer are gradually saturated with ozone molecules, as the tested concentrations for the target gas increase. The desorption of gas molecules from these active centers is made difficult by the low temperature conditions; consequently, active centers on the surface of the sensitive film are more and more occupied, leading to a decrease in the yield of the chemical reaction taking place on the surface of the sensor, which occurs with charge transfer, leading to a change in sensor electrical resistance—captured by the RLC bridge connected to the experimental setup. A smaller resistance variation is reflected by a weaker sensor signal.

Sensor sensitivity *S* to the target gas was defined as

(1) $$S=\frac{R_{gas}-R_{air}}{R_{air}}\times 100\%$$

with *R_gas_* = sensor electrical resistance when exposed to target gas, and *R_air_* = sensor electrical resistance when exposed to carrier gas.

**Figure 8:**
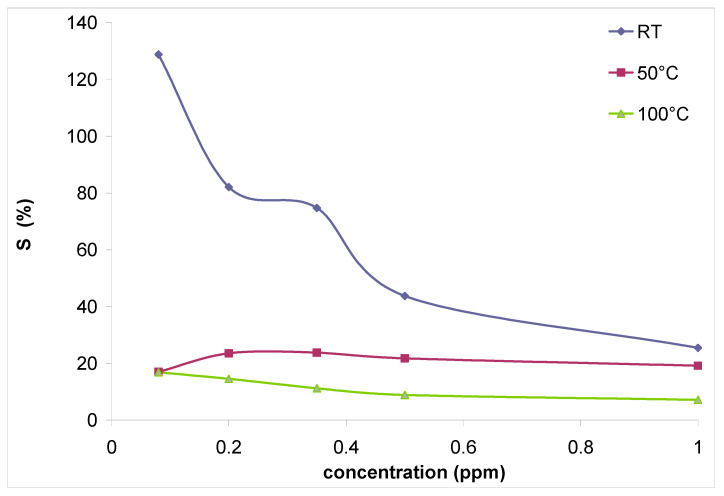
Sensor sensitivity (S) to ozone at different working temperatures.

In Figure 8, we can observe sensor sensitivity to ozone at working temperatures ranging between room temperature and 100 °C. As in the case of sensor response as emphasized in Figure 7, sensor sensitivity to ozone decreases with increasing target gas concentration. Maximum sensor sensitivity is achieved for the lowest ozone tested concentration (0.08 ppm) at room temperature.

Regarding the sensing mechanism involved in ozone detection for this specific case, according to dos Santos Silva et al. [41], for SMOX materials, the interaction between the indium oxide and the ozone molecules (oxidizing gas) leads to ionic species adsorbed on the In_2_O_3_ surface, accompanied by electron consumption, consequently increasing the depletion layer of the oxide material and its electrical resistance (Equation (2)). This was confirmed by the obtained experimental results, in which sensor electrical resistance increases every time ozone is injected in the sensing cell.

(2) $$O_{3}+e^{-}{}_{\left(surface\right)}\overset{}{\to }O_{2(gas)}+e^{-}{}_{\left(surface\right)}$$

According to the same cited work [41], a higher porosity of the sensitive film promotes sensor sensitivity/performance, due to a higher surface area which leads to more available adsorption sites for target gas molecules. The ozone sensor used in this work uses a porous alumina wafer. Together with the surface roughness/porosity characteristics described by the AFM and SEM investigations we can state that the high sensitivity of the investigated sensor at room temperature is in close connection with the porosity of both, the sensitive layer and alumina wafer.

We can state that the sensor signal is clean (very low levels of noise) and its recovery is total, an important sensor characteristic when performing multiple sensing cycles without sensor replacement. For 0.08 ppm ozone in air, similar response values were obtained for 3 consecutive sensing cycles (Figure 5). This sensor characteristic is also linked with sensor sample porosity, according to ref. [41].

**Figure 9:**
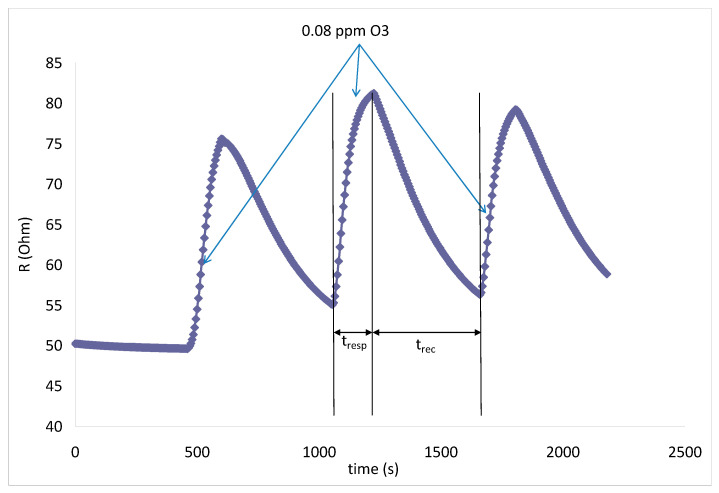
Sensor response/recovery characteristics for 3 consecutive tests with 0.08 ppm ozone in air at room temperature.

In Figure 9, we can observe that reproducible results were obtained for 3 consecutive ozone sensing tests conducted at room temperature for the lowest tested ozone concentration of 0.08 ppm. Fast sensor response, followed by a complete sensor recovery (the electrical resistance value of the sensor returns to the baseline), was also recorded under these testing conditions. The sensor signal was very clean, with negligible levels of noise. Important sensor characteristics were determined using Figure 9, such as sensor response time (t_resp_ = 180 s) and sensor recovery time (t_rec_ = 425 s), both considered as short, implying that the studied sensor is quite responsive to the tested target gas.

Regarding sensor stability, it must be mentioned that sensing measurements were performed several months after sensor fabrication with excellent results (sensor response was ranged between 1.3 and 2.2), so the sensitive film is considered to be chemically stable.

The state of the art regarding SMOX-based ozone sensors, comprised in Table 1, shows that other research groups obtained in some cases successful ozone detection in the ppb range but with higher working temperatures, which involves additional electronics necessary for the detection device, an impediment from the economical perspective [42,43,44,45,46] when mass-production is to be considered.

## 3. Conclusions

A nanostructured In_2_O_3_ film (with 10 layers) obtained by the sol-gel method was used as a sensitive layer in the fabrication of an own-designed chemiresistor, based on an alumina transducer containing Au IDEs and a Pt heating circuit. The deposited sensitive film was characterized using AFM and SEM as continuous, porous and transparent. The sensor responded to low concentrations of ozone (0.08 ppm–1.0 ppm), in compliance with OSHA requirements for the workplace environment (maximum 0.1 ppm for an 8 h exposure time). The sensor response (ranged between 1.3–2.2) was fast (180 s), stable (low-noise and full sensor recovery recorded in 425 s) and reproducible (measurements were performed in triplicates yielding similar ozone detection results) at room temperature, a great advantage from the economical point of view, with no additional heating/electronics being necessary when considering large-scale fabrication. A sensing mechanism was formulated, in agreement with the literature and obtained results, which states that a chemical reaction accompanied by charge transfer occurs on the surface of the In_2_O_3_ film. The sensitive film porosity and also the porosity of the sensor’s alumina wafer both promote gas adsorption and, consequently, sensor performance under low temperature operating conditions. The sensitive layer was considered chemically stable given the fact that the sensing tests were performed with reproducible results several months after sensor fabrication. The low operating temperature of the sensor together with the sol-gel method of obtaining the sensitive layer represents the novelty factor of this research. The good results obtained for the In_2_O_3_-based sensor for ozone detection at room temperature can be used for the future development of a commercial indoor ozone detector.

## 4. Materials and Methods

All chemicals were of analytical grade and used as received without further purification. Indium (III) nitrate hydrate [In (NO_3_)_3_ •xH_2_O], absolute ethanol (CH_3_CH_2_OH) and acetylacetone (CH_3_COCH_2_COCH_3_) were purchased from Sigma Aldrich; ammonia solution 25% EPR was purchased from Labchem.

A solution of 0.2 M In_2_O_3_ was prepared by dissolving indium hydrate nitrate in absolute ethanol at room temperature along with stirring. After dissolving the precursor salt, acetylacetonate (AA) is added dropwise to the solution, as a chelating agent, obtaining a transparent solution. The molar ratio between AA:In (III) was 2:1. After 30 min, 25% ammonia solution was added dropwise and kept under continuous magnetic stirring for an hour. The molar ratio between of NH_3_:In (III) was 2:1. A homogenous, clear and transparent solution was obtained which was stored (for ageing) at room temperature for 6 days.

In order to obtain a good adhesion of the sensitive film to the support, the transducers were previously cleaned with acetone, deionized water and alcohol and dried at room temperature. The obtained precursor solution was deposited onto transducers by dip coating, with an immersion speed of 50 mm/min for 1 min. Ten layers were deposited consecutively, with an intermediate heat treatment applied after each layer at 300 °C for 10 min and a final sensor heat treatment at 350 °C for one hour, a relatively low temperature [55], with a heating rate of 5 °C/minute.

The sensitive layer was deposited onto the alumina transducer in November 2021. Sensing measurements over the fabricated sensor were performed during June–July 2022.

## Figures and Tables

**Table 1:** State of the art regarding ozone detection with SMOX-based sensors.

Materials	Concentration	Operating Temperature (°C)	Sensor Response	LOD	Ref.
Al-doped NiO films	4% O_2_ in plasma	80	2.54	10 ppb	[16]
WO_3_	0.5–2.0 ppm	150	3.9–46.7	-	[47]
ZnO	0.3–5 ppm	200	10.7–26	-	[48]
In_2_O_3_/ZnO		150	14.4	500 ppb	[49]
ZnCo_2_O_4_	80–890 ppb	200	71	890 ppb	[50]
CuWO_4_ nanoparticles	15–50 ppb	250	4.2	15 ppb	[51]
α-Fe_2_O_3_ nanorods	10–570 ppb	150	-	-	[52]
ZnO sputtered films	0.13 ppm	300	-	-	[53]
In_2_S_3_ thin films	40 ppm	160	-	-	[54]
In_2_O_3_ nanostructured films	0.8–1.0 ppm	RT	1.3–2.2	0.08 ppm	This work

## Data Availability

The datasets generated during and/or analyzed during the current study are available from the corresponding author on reasonable request.
